# Autoimmune autonomic ganglionopathy and myasthenia gravis: a case report and review of the literature

**DOI:** 10.1007/s10286-024-01059-8

**Published:** 2024-08-12

**Authors:** Jingwen Yan, Huaxia Yang, Xiaona Jin, Ying Tan, Yuzhou Guan

**Affiliations:** 1 Department of Neurology, Peking Union Medical College Hospital, Chinese Academy of Medical Sciences, Beijing, China; 2 Department of Rheumatology, Peking Union Medical College Hospital, Chinese Academy of Medical Sciences, Beijing, China; 3 Department of Nuclear Medicine, Peking Union Medical College Hospital, Chinese Academy of Medical Sciences, Beijing, China

**Keywords:** Autoimmune autonomic ganglionopathy, Myasthenia gravis, Systemic lupus erythematosus, Nicotinic acetylcholine receptor

## Introduction

Autoimmune autonomic ganglionopathy (AAG) is an autoimmune disease in which antibodies target the nicotinic acetylcholine receptor (AChR) in the autonomic ganglia, resulting in dysfunction of the sympathetic, parasympathetic, and enteric nervous systems [1]. The most commonly reported symptoms of AAG include orthostatic hypotension and gastrointestinal dysmotility, but patients can also experience urinary retention, anhidrosis, dry mouth, erectile dysfunction, and an impaired pupillary light reflex [2]. In patients with intestinal pseudo-obstruction, autopsy can reveal infiltrates of lymphocytes in the gastric and intestinal myenteric plexuses [3]. In 2000, Vernino first proposed AAG and determined its pathogenic antibodies—ganglion nAChR antibodies [4]. The acetylcholine receptor on the autonomic ganglia is a transmembrane pentamer consisting of two α3 subunits and three β4 subunits. Pathogenic antibodies bind mainly to the α3 subunit. A few bind to the β4 subunits [2]. Higher antibody levels (> 0.4 nmol/L) are associated with more severe autonomic dysfunction [5]. Patients with AAG can have extra-autonomic manifestations, including psychiatric symptoms, sensory disturbances, and endocrine disorders [1]. A diagnosis of AAG often suggests the need for screening for potential autoimmune diseases or tumors. Approximately 15% of patients may have tumors (small cell lung cancer, thymoma, or adenocarcinoma) [2]. The first-line treatments are intravenous immunoglobulin (IVIG) and plasma exchange. Additionally, steroids, mycophenolate mofetil (MMF), azathioprine, and rituximab might be effective [6, 7]. Lowering antibody levels to physiological levels through immunotherapy can alleviate clinical symptoms [8].

Myasthenia gravis (MG) is an autoimmune neuromuscular disease characterized by muscle weakness and fatigue. Approximately 80–85% of patients have IgG antibodies specific to the muscle nAChR at the neuromuscular junction. The muscle nAChR has two α1 subunits, along with the β1, δ, and γ or ε subunits. The IgG antibodies target the α1 subunit [9].

We report the case of a patient with antibody-confirmed AAG whose condition improved after immunotherapy. However, after 14 months, the patient developed ocular MG and was found to have antibodies against muscle nAChRs. This case indicates that for patients with AAG, follow-up is important to screen for other autoimmune diseases, including MG.

## Case report

A 49-year-old woman presented with constipation, which she had experienced since February 2022. Her defecation frequency changed from once a day to every 2–3 days, without nausea or vomiting. In March 2022, she developed orthostatic hypotension and experienced two episodes of syncope while standing. She also reported blurry vision in both eyes and reduced sweating in extremities and trunk. In April 2022, she experienced dizziness upon sitting. In the local hospital, the patient exhibited orthostatic hypotension in tilt table test. Her anal sphincter electromyography were normal. A low-amplitude sympathetic skin response was observed in both hands and feet. Anti-nuclear antibodies (ANAs) were 1:1000. Cerebrospinal fluid tests and enhanced MRI of brain and spine were normal. Symptomatic treatment with midodrine was initiated, along with lifestyle interventions. However, the patient continued to experience recurrent syncope upon standing, as well as difficulty in urination, dry mouth, and fatigue. She was admitted to our hospital.

Physical examination revealed significantly elevated blood pressure in the supine position (181/105 mmHg) and low blood pressure upon standing (78/64 mmHg) after 3 min, with decreased heart rate variability (69–74 beats per minute). The right pupil showed a sluggish response to light. There was decreased sensation to pinpricks in a glove-and-stocking pattern. The patient had a history of three episodes of encephalomyelitis from 2017 to 2019, during which ANA positivity was identified. Improvement was observed with steroids. She was treated with azathioprine for 16 months at a dose of 100 mg per day. The patient reported dry mouth and hair loss. Personal and family history were normal.

After admission, ANA with a titer of 1:640 in a homogeneous pattern, anti-nucleosome antibody (12.3 AU/ml), anti-mitochondrial M2 antibody (57.7 AU/ml), SP100, anti-parietal cell antibody (1:320), anti-B2-glycoprotein (18.7 AU/ml), Coombs test (+), and low complement levels (C3, 55.7 mg/dL; C4, 7.7 mg/dL) were observed. Tumor biomarkers and M protein were negative. Enhanced CT revealed incomplete thymic involution without evidence of tumor (Supplementary Fig. 1). An SLE diagnosis, according to the EULAR/ACR 2019 classification criteria, was made on the basis of the presence of clinical (noncicatricial alopecia, nervous system involvement) and immunological criteria (ANA and antiphospholipid antibody positivity and low complement levels). In stomatology and ophthalmology consultations, no evidence of Sjögren’s syndrome was noted. ^131^I-MIBG myocardial scintigraphy showed a normal heart-to-mediastinum ratio (Supplementary Fig. 2). Electromyography showed no abnormalities in nerve conduction velocities or F waves. The patient reported significant weakness and fatigue in addition to obvious orthostatic hypotension, and a repetitive nerve stimulation (RNS) test revealed no decrements in amplitudes to low- or high-frequency stimulation. High titers of ganglion nAChR antibodies (0.48 nmol/L; normal, < 0.02 nmol/L) were observed in radioimmunoassay (RIA).

This patient was diagnosed with AAG. Immunotherapy, including steroid pulse therapy, IVIG, and orally administered cyclophosphamide, was initiated. After discharge, the patient was able to resume household chores and did not experience any further syncope episodes. After 6 months of treatment, with a cumulative dose of 10.5 g of cyclophosphamide, repeat ANA testing yielded negative results, and the patient gradually tapered and discontinued symptomatic medications.

**Fig. 1:**
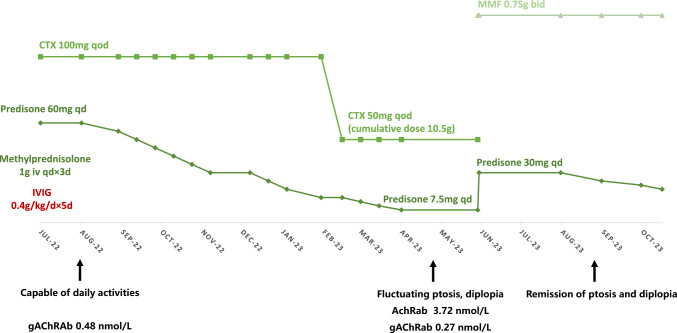
Patient’s disease course and treatments. *CTX* cyclophosphamide, *IVIG* intravenous immunoglobulin, *MMF* mycophenolate mofetil

However, at the ninth month after discharge (April 2023), the patient developed fluctuating ptosis of the right eyelid. One month later, the patient developed diplopia. Physical examination revealed complete occlusion of the right eyelid and limited upward gaze of the right eye. Upward gaze elicited vertical diplopia. RNS showed a decrease in the amplitude of the response of the left facial nerve to low-frequency stimulation. Muscle AChR antibody positivity (3.72 nmol/L) was noted, along with decreased titers of ganglion AChR antibodies (0.27 nmol/L). Cyclophosphamide was discontinued. The patient was prescribed 30 mg orally administered prednisone per day and 1.5 g orally administered MMF per day and resulted in complete remission of symptoms (Fig. 1).

## Discussion

Whether the occurrence of both AAG and MG in a patient is due to cross-immunity or the presence of two pathogenic antibodies is unclear. Ganglion AChRs and muscle AChRs are both members of the nicotinic AChR superfamily. Nicotinic AChRs are a class of pentameric structures formed by the assembly of five subunits into a ring-like structure. The primary immunogenic region recognized by AAG-specific antibodies is the α3 subunit of ganglion AChR; however, MG-specific antibodies recognize the α1 subunit of muscle AChR. Previous studies have shown that the α3 and α1 subunits have 60% structural similarity but differ in immunogenicity (Supplementary Fig. 3). Lennon et al. reported that rabbits passively immunized against the α3 subunit exhibited antibodies against other subunits of acetylcholine receptors, including α1 subunit [10]. In serum samples from patients with MG and AAG, antibody cross-reactivity between AChRs with different α subunits is uncommon and generally weak [11].

Although the structures of the two antibodies are similar and these antibodies can coexist, the combination of AAG and MG is extremely rare. Miglis et al. reported the case of a patient with antibody-confirmed AAG and elevated levels of muscle AChR antibodies who did not meet clinical or electrodiagnostic criteria for MG, suggesting that this patient’s muscle nAChR antibodies seropositivity was a false positive [12]. Lennon et al. [13] reported cases of seven patients who had MG with subacute autonomic failure. Among them, three patients had antibodies against both muscle AChRs and ganglionic AChRs. All three patients had thymomas. The autonomic symptoms were present simultaneously or after the MG symptoms. In contrast, our patient presented with pure autonomic symptoms for 14 months before she developed fluctuating ptosis. This finding indicates that when a patient with immune system abnormalities develops AAG, long-term follow-up is important for assessing the occurrence of other autoimmune diseases, including MG. Li et al. screened for muscle AChR antibodies in 95 patients with AAG and found that only three were positive. Patients carrying both antibodies had no MG symptoms [14]. Nonetheless, there is a case report documenting the coexistence of AAG and MG in a patient with non-small cell lung cancer [15].

Gastrointestinal symptoms in patients with MG and autonomic neuropathy have been reported [3]. Rakocevic et al. reported a case report and review of 12 previous cases of patients with intestinal pseudo-obstruction and MG who were positive for both antibodies. Thymoma may be related to antibodies against neuronal nicotinic receptors in the autonomic ganglia. In our case, the patient’s gastrointestinal symptoms were relatively mild. Gastrointestinal motility endoscopy was suggested, but the patient declined further examination for financial reasons.

In our case, various combinations of autoimmune neurological diseases emerged at different stages of the patient’s disease course, including encephalomyelitis, AAG, and MG. The patient was considered to exhibit antibody epitope spreading and immune generalization in the context of an immune background and incomplete thymic involution. Following treatment with rituximab and thymectomy can be considered.

## Conclusion

In patients with acute/subacute onset of autonomic dysfunction, ganglionic AChR antibody testing is recommended. Ganglion antibody titers can be monitored to evaluate the effectiveness of immunotherapy. Patients with various autoimmune antibodies should be screened for underlying tumors and autoimmune diseases. Understanding the structure and function of ganglion AChRs and muscle AChRs in patients with coexistent AAG and MG is important.

## Supplementary Information

Below is the link to the electronic supplementary material.

Supplementary file 1 Enhanced CT revealed incomplete thymic involution (JPG 49 KB)

Supplementary file 2^131^I-MIBG myocardial scintigraphy was used to clarify the status of sympathetic nerve innervation of the heart. After intravenous injection of^131^I-MIBG, local imaging of the mediastinum was performed at 15 mins and 4 h, showing the accumulation of the tracer in the heart region. The 15-min heart-to-mediastinum ratio was 2.16, and the 4-h H/M ratio was 2.94. These heart-to-mediastinum ratios were normal (JPG 70 KB)

Supplementary file 3 Illustration of the muscle nAchR and ganglion nAchR in the MG and AAG. Created with BioRender.com (PDF 106 KB)
