# Supplementary figures and images for: Autoimmune autonomic ganglionopathy and myasthenia gravis: a case report and review of the literature
Source: Clin Auton Res. 2024 Aug 12;35(1):149–52. doi: 10.1007/s10286-024-01059-8 (PMC11937030; doi:10.1007/s10286-024-01059-8)

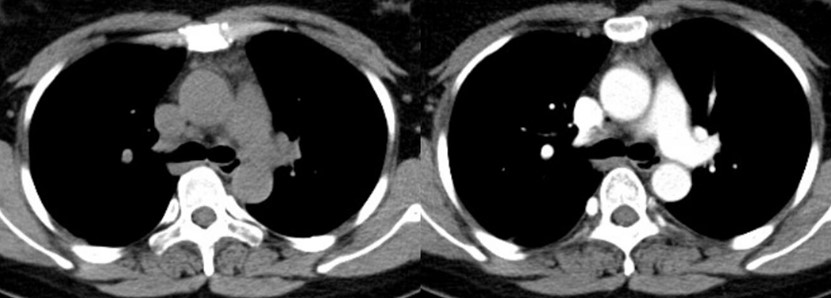

Supplement: Supplementary file 1 — Enhanced CT revealed incomplete thymic involution (JPG 49 KB) [file 10286_2024_1059_MOESM1_ESM.jpg]

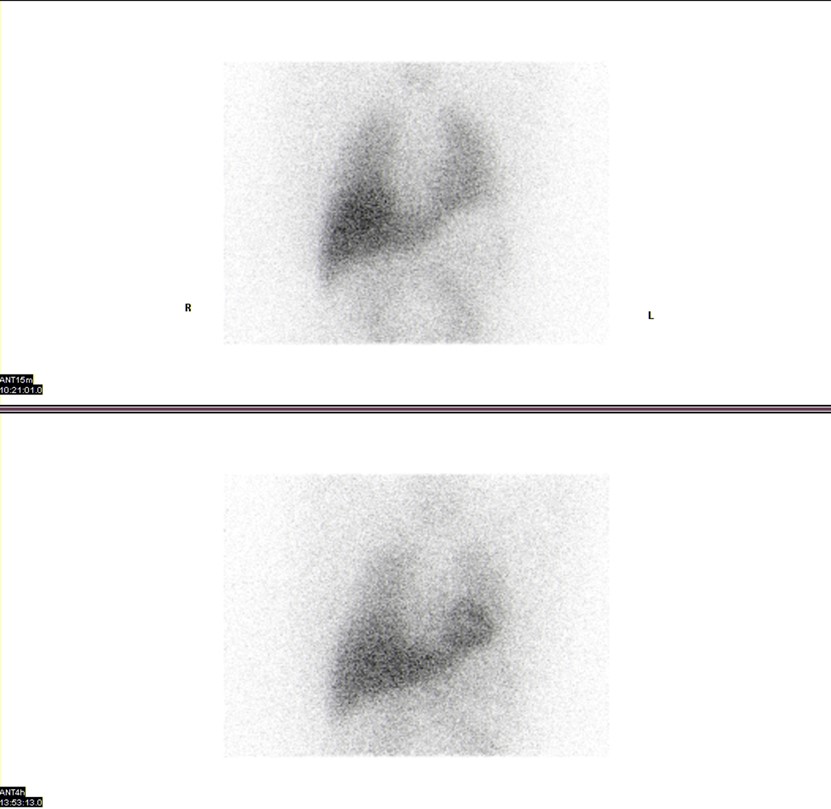

Supplement: Supplementary file 2 — 131I-MIBG myocardial scintigraphy was used to clarify the status of sympathetic nerve innervation of the heart. After intravenous injection of131I-MIBG, local imaging of the mediastinum was performed at 15 mins and 4 h, showing the accumulation of the tracer in the heart region. The 15-min heart-to-mediastinum ratio was 2.16, and the 4-h H/M ratio was 2.94. These heart-to-mediastinum ratios were normal (JPG 70 KB) [file 10286_2024_1059_MOESM2_ESM.jpg]

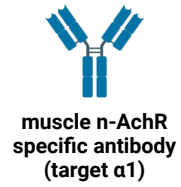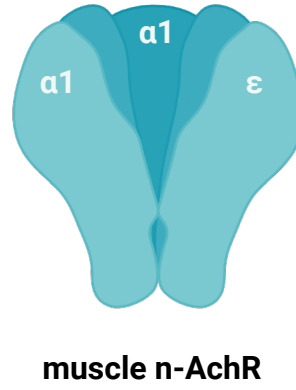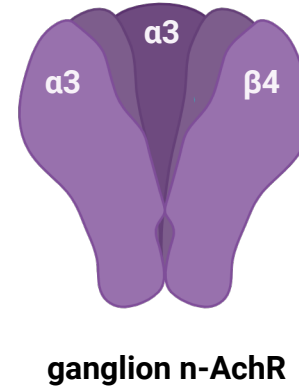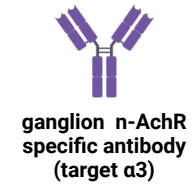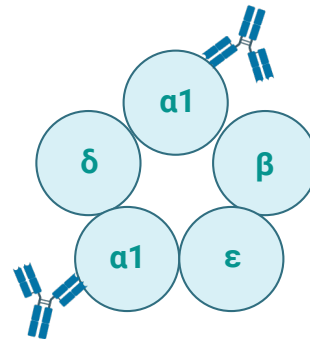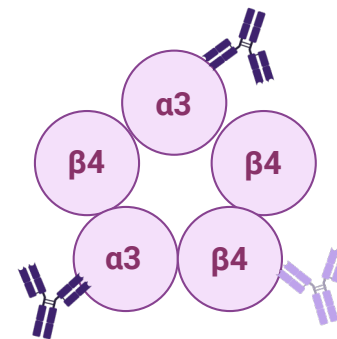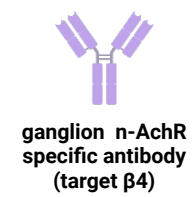

Supplement: Supplementary file 3 — Illustration of the muscle nAchR and ganglion nAchR in the MG and AAG. Created with BioRender.com (PDF 106 KB) [file 10286_2024_1059_MOESM3_ESM.pdf]
